# EEG-Based Estimation on the Reduction of Negative Emotions for Illustrated Surgical Images

**DOI:** 10.3390/s20247103

**Published:** 2020-12-11

**Authors:** Heekyung Yang, Jongdae Han, Kyungha Min

**Affiliations:** 1 Division of Software Convergence, Sangmyung University, Seoul 03016, Korea; yanghk@smu.ac.kr; 2 Department of Computer Science, Sangmyung University, Seoul 03016, Korea

**Keywords:** emotion, EEG, DEAP, CNN, surgery image, disgust

## Abstract

Electroencephalogram (EEG) biosignals are widely used to measure human emotional reactions. The recent progress of deep learning-based classification models has improved the accuracy of emotion recognition in EEG signals. We apply a deep learning-based emotion recognition model from EEG biosignals to prove that illustrated surgical images reduce the negative emotional reactions that the photographic surgical images generate. The strong negative emotional reactions caused by surgical images, which show the internal structure of the human body (including blood, flesh, muscle, fatty tissue, and bone) act as an obstacle in explaining the images to patients or communicating with the images with non-professional people. We claim that the negative emotional reactions generated by illustrated surgical images are less severe than those caused by raw surgical images. To demonstrate the difference in emotional reaction, we produce several illustrated surgical images from photographs and measure the emotional reactions they engender using EEG biosignals; a deep learning-based emotion recognition model is applied to extract emotional reactions. Through this experiment, we show that the negative emotional reactions associated with photographic surgical images are much higher than those caused by illustrated versions of identical images. We further execute a self-assessed user survey to prove that the emotions recognized from EEG signals effectively represent user-annotated emotions.

## 1. Introduction

From the early days of brain science, many researchers have studied negative human emotions that come from surgical images showing blood, injection, and injury (BII). Usually, the negative emotion caused by surgical images is classified as fear and disgust. Even though emotional responses can be different according to the subject’s experience, knowledge, and personality, the emotional responses are located in close distances when mapped on widely used Russell’s emotional coordinate [1].

Ordinary people rarely have the chance to view surgical images. However, there are certain situations in which people should carefully inspect surgical images. For example, when we or our family members face surgical operation, surgeons explain the process of the operation by showing surgical images. Even though we feel very negative emotions in response to the images, we still have to study them very carefully to understand the procedure. In cases such as this, the negative emotional response to surgical images can prevent people from understanding necessary surgical procedures.

Some studies have tried to reduce impact of the image by simplifying the color of the images through image abstraction schemes [2,3]. They employed existing image abstraction techniques on surgical images to produce illustrated expressions in order to reduce the negative affective responses. Even though many of the existing image abstraction techniques fail to present the details of the surgical images, some surgeons participating in these studies recommend the illustrated surgical images for explaining the images to ordinary people or training students [4,5].

Even though several image abstraction algorithms such as [4,5] have demonstrated effectiveness for abstracting surgical images, it is difficult to retain the diverse and fine details of human organic structures which is especially important for educational purposes while reducing negative emotional response. It would take a lot of cost and effort to develop an algorithm that satisfies the above requirements. Before undertaking such an effort, we need objective evidence that the illustrated surgical images can reduce the severity of negative emotional reactions to photographic surgical images. Unfortunately, the works of Besancon et al.’s [2,3] lack such an evidence, as they did not include quantitative study such as EEG-based emotion measurement on the emotional reactions comparing abstracted surgical images from original images.

We argue that objective and quantitative strategies including biosignal-based methods are needed to confirm the hypothesis that illustrated surgical images can reduce negative emotional reactions. The cost of developing an automatic algorithm that produces illustrative representations of surgical images with preserving sufficient details would be surprisingly expensive; before deciding whether to develop such an algorithm, we need concrete evidence that supports our hypothesis. Among the various kinds of biosignals, the electroencephalogram (EEG) is very widely used to measure emotional responses. Recently, many deep learning-based methods employing EEG have been presented [6,7,8,9,10,11,12,13,14,15,16,17,18].

We employ an EEG-based emotion recognition framework in order to present a quantitative measure of the difference in emotional reactions to illustrated surgical images and photographic surgical images. To this end, we employ professional scientific illustrators to produce illustrations of surgical images, and execute a user study with a deep learning-based emotion recognition model with EEG biosignals. Our study seeks to provide a confidence that the negative emotional reactions caused by surgical images can be reduced through abstraction via illustration.

We employ two groups of participants who view photographic surgical images and illustrated surgical images, respectively. Their emotional responses are measured through an EEG capturing device and processed with a deep learning-based emotion recognition model. To show the effectiveness of the model, we additionally execute a nine-metric user survey for the participants, then compare those results with those of the model. From the results of this experiment, we suggest that the illustrated surgical images successfully reduce the negative emotional responses caused by their photographic counterparts.

## 2. Related Work

### 2.1. Emotional Reactions to Surgical Images

Many researchers have studied human emotional reactions on BII (blood, injection, and injury) scenes including body mutilation [19], surgical procedures [20,21], blood drawing [22], open-heart surgery [23], and surgical amputation [24]. Other studies presented human reactions to repelling scenes including homicide scenes [25], spiders [21], vomit [23], and dirty toilets [26]. To estimate human reactions to these scenes, most studies employed either estimation of physical reactions or subjective methods. For estimating physical reactions, they examined heart rate [19,23], facial expression [27], electromyography [23,28], skin conductance [28], neural activation by fMRI [26], eye tracking [29], and visuomotor processing [30]. The subjective methods include user survey [20,21], rate of refusal to watch [23], and experienced vasovagal symptoms [22]. Even though these schemes are used to estimate human reactions to repelling scenes including surgery images, Cisler et al. found that there is no universal scheme to consistently estimate human reactions [31].

Surgical images incur various human reactions including anxiety, fear, disgust, and vicarious pain [31,32]. Among them, fear and disgust are suggested to be the most prominent. Cisler et al. and Olatunji et al. reported disgust as the most representative emotion [31,33]. Their reason is that fear is an emotion of avoiding danger, which is not induced by watching images of bodily injury or surgery. Chapman and Anderson classified blood-injury disgust as a subtype of physical disgust [34]. Olatunji et al. further divided blood-injury disgust into contamination disgust and animal-reminder disgust, where animal-reminder disgust is defined as the reminder of one’s mortality and inherent animal nature [23].

### 2.2. CNN-Based Emotion Recognition from EEG

From the great success of AlexNet [35] and VGGNet [36] in image classification, deep convolutional neural networks are employed in various EEG analytic tasks.

Tang et al. [6] proposed an early deep CNN-based classification model for single-trial EEG. Their model, which is composed of five layers, is applied to classify motor imagery of left and right hand movement. The model recorded F1 scores of 87.76% 86.64% for classification of motor imagery. While the performance of the model does not show significant improvement over conventional hand-crafted feature-based models, this study has shown the promise of CNN for EEG recognition.

Schirrmeister et al. [7] presented a deep CNN to analyze EEG biosignals. Their model is composed of four blocks, each of which executes convolution and max pooling operations. The first block is distinguished from the others, as it executes spatial filtering between the convolution and max pooling operations. The result of fourth block is linearly classified to four soft max units including left hand, right hand, feet, and rest. They also visualized the features of their CNN to analyze the relationships between the features and EEG signals.

Li et al. [17] presented a hybrid model of convolutional neural network and recurrent neural network to recognize emotions from a multi-channel EEG dataset. As a preprocessing of their data, they employ wavelet and scalogram transform to encapsulate multi-channel neuro-signals into grid-like frames. Their model extracts task-related features and mines correlation between channels, incorporating contextual information. They demonstrate their accuracy by estimating valence and arousal.

Salama et al. [8] presented a 3-dimentional CNN approach for recognizing emotions from multi-channel EEG signals. Their model is constructed using two convolutional layers and two max pooling layers, followed by a fully connected layer. The 3D representation of EEG data is fed into a data augmentation phase, which improves the performance of their 3D CNN model. The simple structure of their model leaves many aspects to be improved. They achieved 87.44% accuracy for valence and 88.49% for arousal.

Moon et al. [9] applied CNN models for EEG-based emotion recognition. They tested three CNN models: CNN-2, CNN-5, and CNN-10, which are distinguished by the number of convolutional layers. The CNN-2 model has one convolutional layer followed by one max pooling layer; CNN-10 has five convolutional layers followed by five max pooling layers. Among the three models, CNN-5, which has three convolutional layers and two max pooling layers, shows the best accuracy for PSD, PCC, and PLV features.

Chiarelli et al. [10] presented a hybrid framework to construct a brain-computer interface using EEG and infrared spectroscopy (fNIRS). They employed DNN, which recorded unprecedented classification outcomes, for their framework. They performed a guided left and right hand motor task on 15 participants. The left-to-right classification accuracy of the DNN was estimated and compared to a stand-alone EEG and fNIRS. The results of their multi-modal recording and DNN classifier significant improvement over the state-of-the-art techniques.

Lawhern et al. [11] presented an EEGNet, a DNN-based approach for constructing brain-computer interface using EEG biosignals. Their EEGNet has three blocks of layers: Conv2D, DepthwiseConv2D, and SeparableConv2D. In this network, they introduce depthwise convolution and separable convolution to process the EEG signals effectively. They tested their method on three datasets: P300 Event-related Potential, Feedback Error-related Negativity, and Movement-related Corticial Potential. The results demonstrate EEGNet’s improved performance over reference algorithms.

Croce et al. [12] applied a CNN model to a large dataset of independent component (IC)s extracted from multi-channel EEG and magnetoencephalographic (MEG) signals. Their aim was to classify brain IC and artifactual IC from the biosignals. The EEG, MEG, and combined EEG + MEG signals were processed through a CNN model to compare its accuracy of classification with state-of-the-art models; their classification accuracies reached 92.4% for EEG, 95.4% for MEG and 95.0% for EEG + MEG.

Yang et al. [13] proposed a CNN-based approach to recognize valence and arousal from unstationary EEG signals. Their model has a multi-column structure of independent modules, each of which was designed using DenseNet [37]. The independent decisions from the modules were merged using a voting strategy to make a final decision. Their model was trained and optimized using DEAP dataset, and applied to distinguish the emotional responses between photographs and artwork images [38] and to verify the influence of contrast on valence [39].

### 2.3. RNN-Based Emotion Recognition from EEG

The spatio-temporal aspect of EEG biosignals invites the use of a recurrent neural network (RNN), which is known as an effective model for processing time-serial data, to analyze EEG signals.

Khosrowabadi et al. [14] presented a biologically inspired feedforward neural network (ERNN), which has six layers, to recognize human emotions from EEG biosignals. The ERNN model employs a serial-in/parallel-out shift register to simulate the short term memory of emotion. This model with a radial basis function shows very competitive accuracy compared with other feature extraction methods.

Alhagry et al. [16] presented an RNN-based emotion recognition model from EEG biosignals. Their model has two long short term memory (LSTM) layers, one dropout layer and one fully connected layer. Since the EEG biosignal captured from subjects watching a movie clip has a time-serial property, the RNN structure demonstrates competitive accuracy in emotion recognition. Their model showed 85.65% accuracy for arousal and 85.45% for valence.

Soleymani et al. [15] employed an LSTM RNN with conditional random fields to trace the emotions captured from EEG biosignals of subjects watching video. They also captured facial expressions of the subjects. The combination of EEG biosignals and facial expressions was able to provide adequate information for emotion recognition.

Xing et al. [18] presented a framework consisting of a linear EEG mixing model and an LSTM RNN model. For EEG mixing, they employed stack auto encoder (SAE), which is similar to the standard auto encoder; the difference lies in the processing of source signals, which are separated by brain region. The EEG source signals processed by SAE are fed into the LSTM RNN model, which then extracts features from them. This model achieved 81.10% accuracy for valence and 74.38% for arousal.

## 3. Overview of Our Framework

Our assumption is that the negative emotions caused by photographic surgical images would hinder non-professional people in understanding necessary information conveyed by the images. Since these people are not trained with surgical images, the negative emotions such as fear and disgust arise at their first glance of the images. To reduce negative emotion and to increase the understanding of the information conveyed by surgical images, anatomy textbooks tend to use illustrations, rather than photographs. From this point, we build our assumption that illustrated surgical images evoke less negative emotion from ordinary people than photographic surgical images.

**Figure 1:**
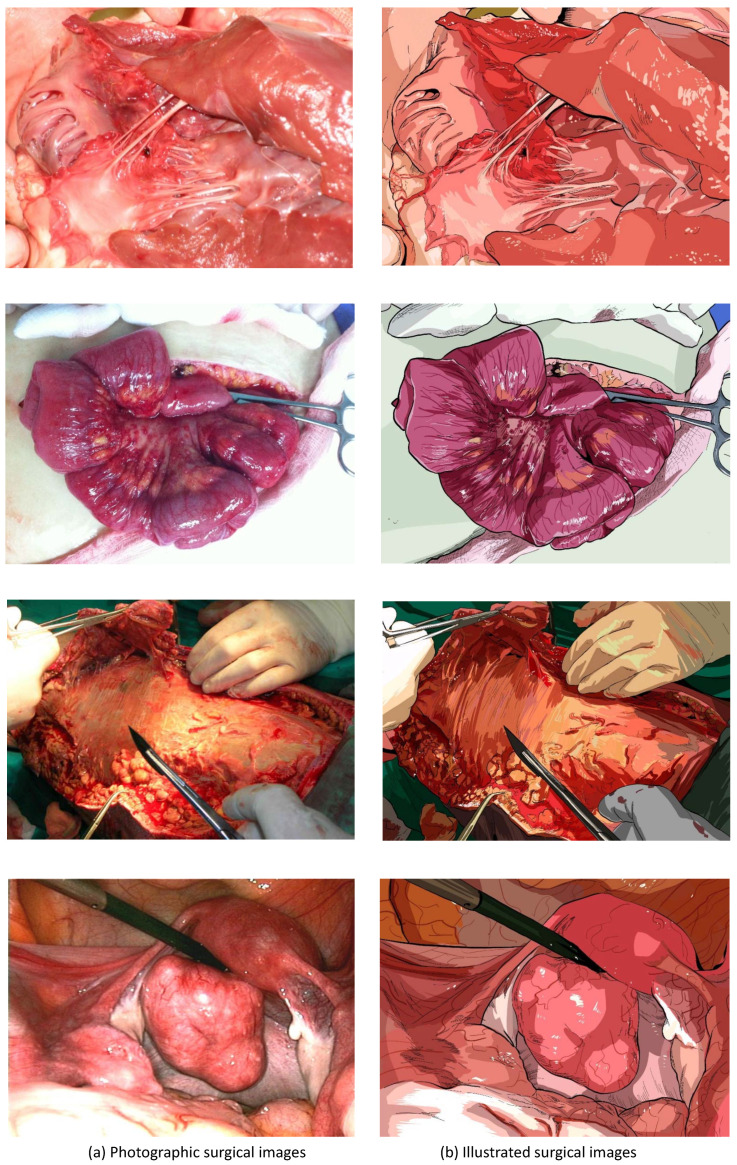
The first comparison of photographic surgical images and illustrated surgical images.

**Figure 2:**
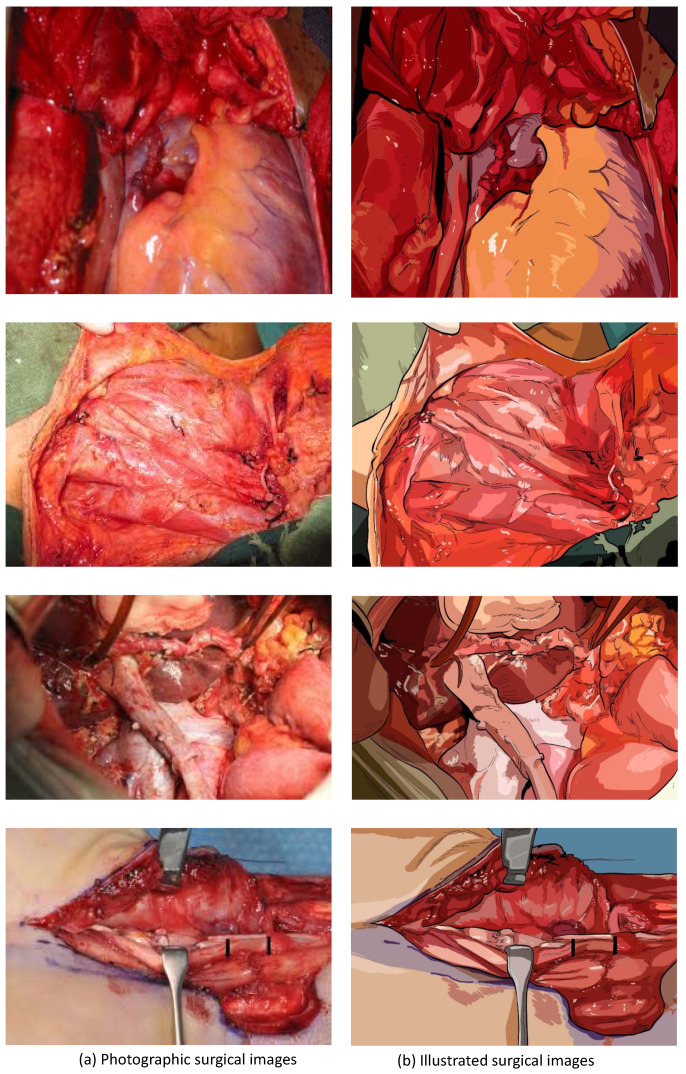
The second comparison of photographic surgical images and illustrated surgical images.

**Figure 3:**
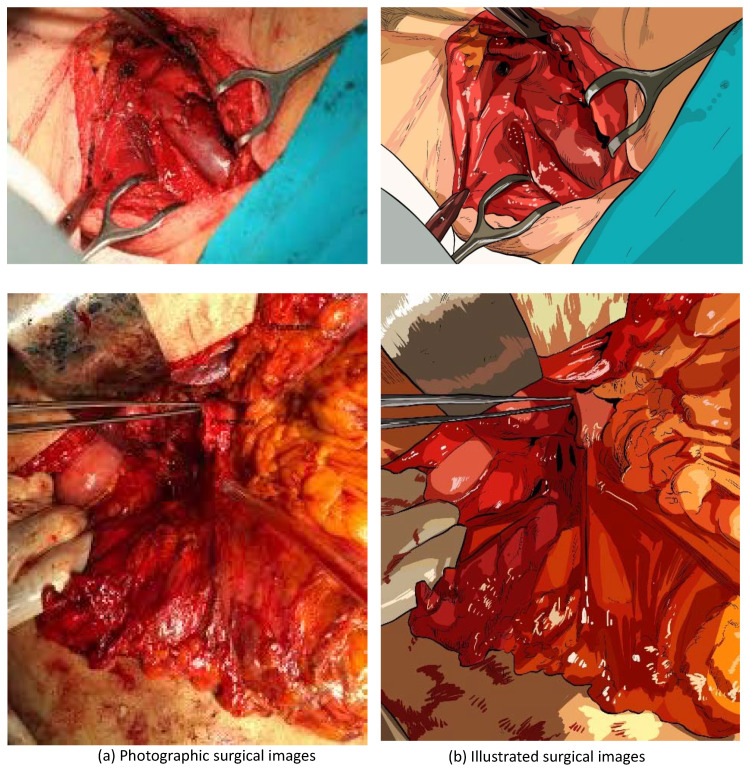
The third comparison of photographic surgical images and illustrated surgical images.

To prove our assumption, we chose 10 photographic surgical images and produced their illustrated versions by hiring professional scientific illustrators. The photographic surgical images and their illustrated counterparts are presented in Figure 1, Figure 2 and Figure 3. Before producing the illustrations, the illustrators were instructed to preserve as much fine details as possible. The target images were collected from various sources and are released under ’fair use’ purpose.

From the ten pairs of photographic and illustrated surgical images, we hired 40 participants to record their emotional reactions to both photographic surgical images and illustrated images. To record their emotional reactions, we employed two different processes. One was the usage of EEG biosignals; the EEG signals captured from participants were processed through a deep multi-channel emotion recognition model [13] and quantized into valence and arousal scores. The other process was a self-assessed user survey. The participants were given a 9-metric survey to record their valence and arousal.

**Figure 4:**
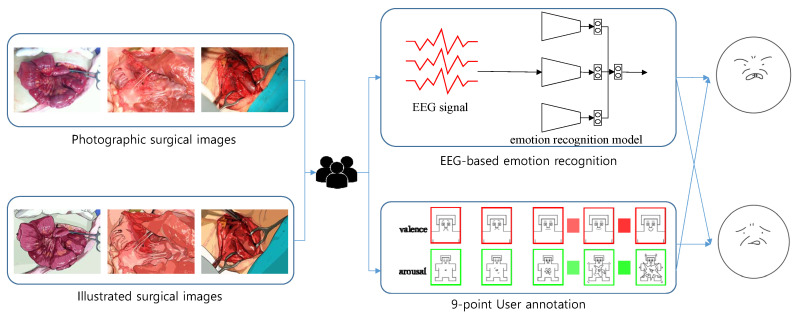
Overview of our framework.

The 40 participants were randomly partitioned into two groups. The first group underwent the above processes for photographic surgical images, while the second worked with illustrated images. The results of these two groups are analyzed and discussed; the outline of this study is illustrated in Figure 4.

## 4. Deep Emotion Recognition Model

### 4.1. Structure of the Model

In this section, we describe our deep emotion recognition model, which was presented in our previous study [13]. Our model is based on a multi-column structure: five independent modules process the EEG signal and make estimations of valence and arousal. Results from these modules are ensembled into concerted valence and arousal scores, effectively recognizing emotional responses.

### 4.2. Dataset Preparation

**Figure 5:**
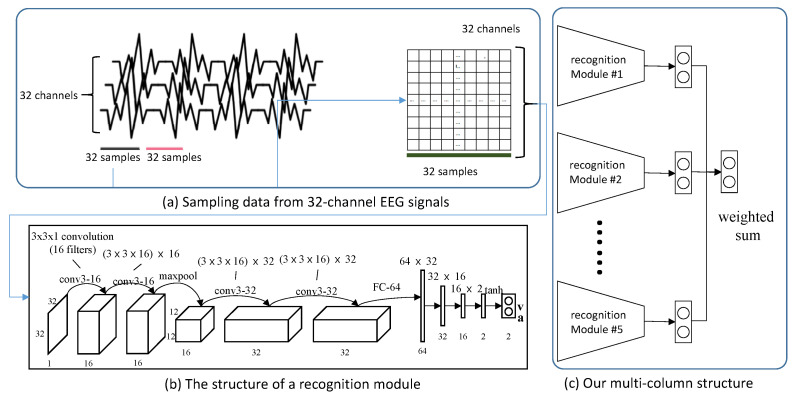
Structure and dataflow of our model, which is constructed according to [13].

For the training of our model, we employ the DEAP dataset [40], one of the most widely used EEG datasets. The DEAP dataset consists of preprocessed EEG signals and their corresponding labels which describe emotional states. As instructed by the original authors [40], and similar to our previous study [38], we downsample the EEG signal in DEAP to 128 Hz and process it with a 4.0–45.0 Hz band pass filter. Therefore, we extract 128 × 60 samples from a trial for 40 channels of the dataset. Among the 40 channels, we exclude 8 for normalization and employ 32 channels for the input of our model. For each input channel, we prepare 32 consecutive samples for an input for each module of our model, effectively creating 32 × 32-sized input data. Figure 5 (a) illustrates the sampling process of EEG data, (b) shows the structure of a recognition module, and (c) shows the overall multi-column structure of the model.

### 4.3. Model Training

The DEAP dataset is constructed using the EEG signals captured from 32 participants. We segment the dataset into three groups: training, validation and test. Out of the 32, EEG signals from 22 participants are used for training, validation and the remaining 5 for test. Each participant executed 40 experiments. Therefore, the numbers of the EEG signal data for training, validation, and test are 880, 200, and 200, respectively.

## 5. Implementation and Experiment

### 5.1. Implementation

We implemented our emotion recognition model with Pytorch library on a machine with an Intel Core i7 CPU, 64 GB main memory, and nVidia GTX 2080TI GPU. We employed LiveAmp32 with LiveCap [41], which supports 32 channels following a standard 10/20 system [42].

### 5.2. Preparation of Surgical Images

While preparing our dataset of surgical images, we surveyed various open emotional image datasets including IAPS [43], GAPED [44], NAPS [45], CAPD [46], SMID [47], ISEE [48] and COMPASS [49]. Some of the datasets are specialized for scary images (SFIP) [50], disgusting images (DIRTI) [51], natural disaster images (NDPS) [52], and adult images (BAPS-adult) [53]. However, there was no existing dataset dedicated to surgical images; therefore, we collected several “fair-use” images from various sources for our experiment.

### 5.3. Preparing of User Annotation

**Figure 6:**
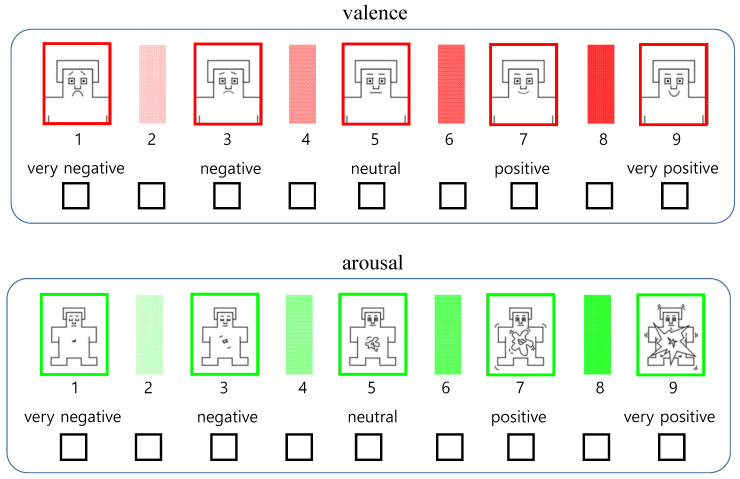
Nine-point metric for user annotation.

For user annotation of emotional responses, we presented participants with a nine-point metric separated into valence and arousal. They were asked to mark the metric for the photographic and illustrated surgical images they saw. The leftmost point means very negative reaction, which matches to −1 in EEG-based estimation, and the rightmost point means very positive reaction, which matches to 1. The mid-point signifies a neutral reaction, which matches to 0. The nine-point metric form for user annotation is presented in Figure 6.

### 5.4. Experiment

**Figure 7:**
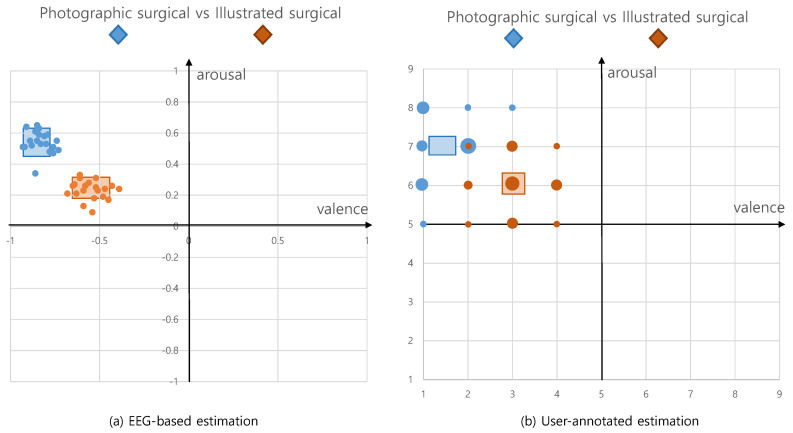
Results plotted in Russell’s emotion circumplex model.

**Figure 8:**
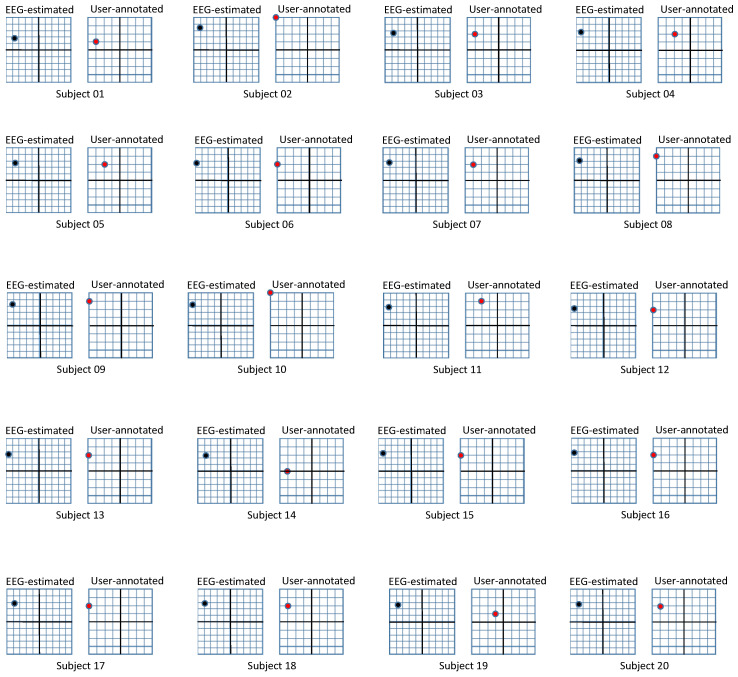
Results from photographic surgical images. Subjects 01∼20 participated in the experiment for photographic surgical images. The left box, which corresponds to EEG-based recognition has a range of (−1,1)×(−1,1), while the right box corresponding to user-annotated emotion has a range of (1,9)×(1,9). The x-axis of each box represents valence and the y-axis represents arousal.

**Figure 9:**
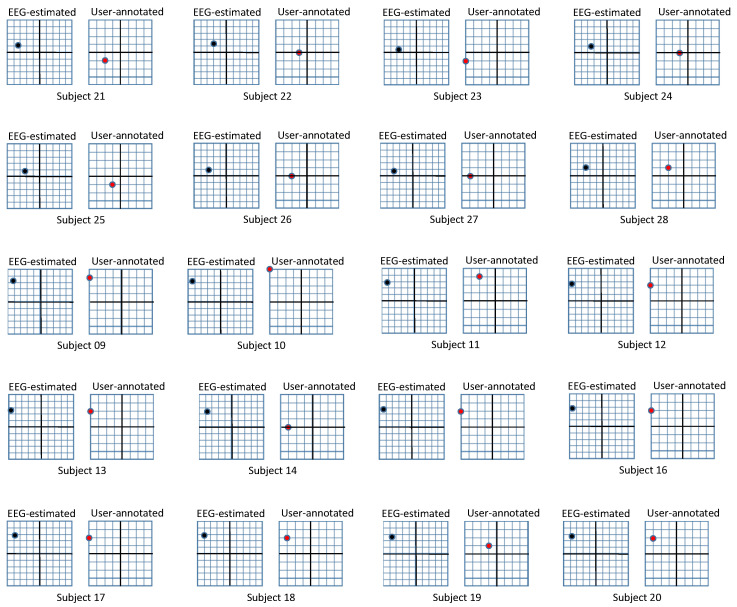
Results from illustrated surgical images. Subjects 21∼40 participated in the experiment for illustrated surgical images. The left box, which corresponds to EEG-based recognition has a range of (−1,1)×(−1,1), while the right box corresponding to user-annotated emotion has a range of (1,9)×(1,9). The x-axis of each box represents valence and the y-axis represents arousal.

For the experiment, we hired 40 participants and separated them into two groups: group1 watched photographic surgical images and group2 watched the illustrated versions. The characteristics of the two groups are suggested in Table 1. In the case of a participant watching both photographic and illustrated surgical images in a short timeframe, the emotional response from the images they watched first can affect that from the images that they watched later. This was our motivation for employing disjoint groups. We address the issue that personal differences between participants may affect their emotional responses by increasing the number of participants. Before participants watched the images, we explained what they were about to see and allowed withdrawal from the experiment. The participants were asked to watch a 100 s movie clip, wich each image lasting 10 s. In the first round of our experiment, we extracted EEG biosignals from the participants for objective responses. We performed the second round by asking the participants to mark their valence and arousal on a 9-point metric for subjective responses. To avoid the diminishment of emotional reactions in the second round, the participants were instructed to remember their emotions during the first round and mark those. The results of both rounds of the experiment on each group are illustrated in Table 2 and Figure 7. We further visualize the comparison of personal responses for EEG-based and user-annotated emotion in Figure 8 and Figure 9.

## 6. Analysis

We have two research questions regarding our experiment.

- RQ1 Are emotional responses from illustrated surgical images discernably less negative than those from photographic surgical images?
- RQ2 Is our emotional recognition model reliable? In other words, is there sufficient evidence that the emotions recognized by our model resemble self-assessed ones?

### 6.1. Analysis 1: t-Test

We have set up our null hypothesis for RQ1 as follows:

- $H_{0}$ There is no notable difference between emotional responses from photographic surgical images and illustrated images.

To answer RQ1, a *t*-test is executed between group1 who watch photographic surgical images and group2 who watch illustrated surgical images. The *p* values of this *t*-test are presented in Table 3. According to the very small *p* values in Table 3, we can reject $H_{0}$ in favor of the alternative hypothesis.

Another *t*-test regarding the emotions estimated by EEG and the emotions annotated by users, leads us to answer RQ2. RQ2’s null hypothesis is:

- $H_{1}$ There is no notable difference between EEG-based assessment and user-annotated approach.

The *p* values for this second *t*-test are presented in Table 4. For group1, who watched photographic surgical images, the valence and arousal estimated from EEG biosignals and annotated by users are very closely related; the strong negative emotion recognized from photographic surgical images is consistent regardless of the recognition scheme. However, for group2, who watched illustrated surgical images, the valence is closely related, while the arousal is not. Therefore, we cannot reject $H_{1}$ except in case of arousal. We assume that pictures of organs, blood, and flesh effect similar negative emotions in viewers, even though they are reduced by the illustrated representation. Therefore, the different approaches for estimating emotions show consistently similar valence scores; for arousal, however, ordinary people have only rarely seen surgical images even, even in their illustrated form. Users who have watched illustrated surgical images in the first round of the emotion recognition experiment through EEG biosignals may pay less attention in the second stage experiment using user annotation. Therefore, the arousal values for illustrated surgical images in user annotation can be lower than those in EEG-based estimation.

### 6.2. Analysis 2: Effect Size

We estimate the effect size by calculating Cohen’s *d* values for the pairs of the emotions. The formula for Cohen’s *d (X, Y)* is suggested as follows:

$$d\left(X,Y\right)\;=\;\frac{Exp(X)-Exp(Y)}{SD_{pooled}},$$

where Exp(X) and Exp(Y) are the mean values of the distributions *X* and *Y*, respectively, and $SD_{pooled}$ is the pooled standard deviation of *X* and *Y*.

We estimate Cohen’s *d* to measure the difference between the emotional reactions of photographic and illustrated surgical images in the following four combinations in Table 5:

(i) the valence estimated by EEG between photographic and illustrated surgical images,
(ii) the arousal estimated by EEG between photographic and illustrated surgical images,
(iii) the valence estimated by user-annotation between photographic and illustrated surgical images,
(iv) the arousal estimated by user-annotation between photographic and illustrated surgical images.

The Cohen’s *d* values for these four matches are greater than 0.8, which denotes that the effect size is very large.

We also estimate Cohen’s *d* to measure the difference between the emotions estimated by EEG and the emotions estimated by user-annotation in the following four combinations in Table 6:

(i) the valence for photographic surgical images estimated by EEG biosignal and user annotation,
(ii) the arousal for photographic surgical images estimated by EEG biosignal and user annotation,
(iii) the valence for illustrated surgical images estimated by EEG biosignal and user annotation,
(iv) the arousal for illustrated surgical images estimated by EEG biosignal and user annotation.

The Cohen’s *d* values for cases (i)∼(iii) are less than 0.23, which denotes that the effect size is small, and the *d* value for case (iv) implies there is relatively larger effect size. The reason case (iv) has a medium effect size can be described as similar to the reason results from the case (iv) in the prior *t*-test are more weakly related.

## 7. Discussion

### 7.1. Discussion 1: Comparison of Performances

In the relevant literatures, many models, including conventional machine learning techniques or deep learning techniques, have been employed to estimate valence and arousal from EEG signal. According to [13], models using machine learning-based approaches such as SVM or decision tree show 71.66% average accuracy for valence and 69.37% for arousal, while the models using deep learning schemes such as CNN or RNN show 81.4% for valence and 80.5% for arousal. Clearly, emotion recognition schemes based on deep learning techniques outperform those based on conventional machine learning techniques. The accuracy of our model is compared to that of several important existing studies that estimate emotion through valence and arousal, as shown in Table 7.

### 7.2. Discussion 2: Increase of Valence

Our experiment reveals that the valence estimated from illustrated surgical images is significantly higher than the valence from photographic surgical images. We assume that the unpleasant feelings from photographic blood and flesh are decreased by substituting the color of photographic blood and flesh with a similar color that has higher saturation or intensity. Classic artistic media, such as pencil or watercolor brush, produce similar effects. It is also notable to invoke the conclusion of the work of Yang et al’s work [38] that the artwork images induce higher valence than photographs. Since the illustrated surgical image can be regarded as a kind of artwork, the increased valence for the illustrated surgical image reinforces the conclusion of [38].

The change in valence in our study, however, is greater than that in [38]. We assume that emotional reactions to surgical images generally more negative: fearsome or disgusting. Therefore, the increase of valence between the surgical image and its illustrated version is greater than the increase of valence between a photograph and an artwork image when the image itself is neutral.

### 7.3. Discussion 3: Decrease of Arousal

Our result shows a decrease in arousal for the illustrated surgical images compared to the photographic surgical images. Since photographic surgical images are distinguished from other images, their engendered arousal is very great. The reason for the photographic surgical images showing higher arousal is reasoned to be that seeing flesh and blood usually occurs only in very frightening or alerting situations. The decrease in arousal for the illustrated images can be explained by the fact that the realistic colors of flesh and blood are converted to less threatening colors frequently seen in animations or cartoons. The simple and friendly color of the illustrated flesh and blood reduces the sense of actual alert or frightening, which results in the decrease of arousal.

### 7.4. Discussion 4: Evaluation from a Surgeon

**Figure 10:**
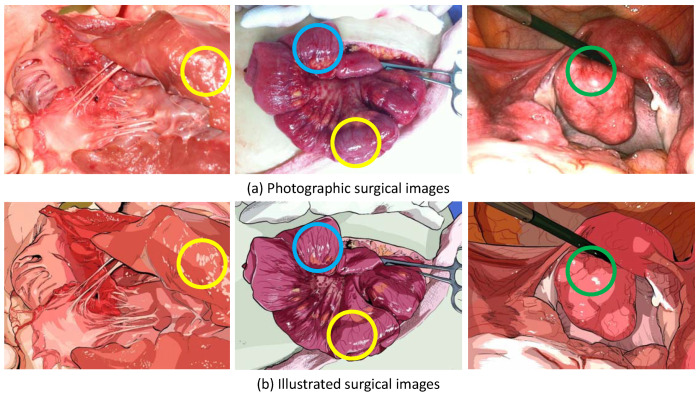
The marks drawn by a surgeon for the evaluation of the illustrated images.

We have asked a surgeon to evaluate the illustrated surgical images. The surgeon marked some regions of the photographic and illustrated surgical images and suggested the following opinions:

(1) Color transform of the illustrated images is reasonable. Replacing vivid colors such as red and violet by less vivid colors can help reducing negative reactions from the people who do not have an experience in the surgical images
(2) In the illustrated versions, the reflections on the surface of organs are illustrated as a narrow spot with higher brightness (the yellow circles in Figure 10). Since the spots on organs can be from some disease or from the reflection, the reflections should be illustrated in different style.
(3) The blood vessels, which play important role in many diagnosis cases, are not illustrated in a consistent way. In some figures, they are preserved in a very salient way (the blue circles in Figure 10), and in others, they are omitted (the green circles in Figure 10). Presenting details such as blood vessels should be expressed in a consistent way.

As a conclusion, the surgeon suggested a positive answer for using the illustrated images to reduce negative emotional reactions from ordinary people. However, he suggested several points to improve for educational or professional purposes.

### 7.5. Discussion 5: Limitations

The limitation of this study is that we have not taken into account the opinions of experts, such as surgeons or pathologists in producing the illustrative surgical images. Surgeons may provide productive insights in the illustration of surgical images, for example, which fine details are important and must be kept. They may also be able to suggest proper colors to replace the original colors of flesh and blood.

This limitation of this study can be addressed in two points. Similar studies [2,3] hired a small group of surgeons to confirm that the illustrated surgical images could be used for communication and education. In another point, we collected a series of opinions from a surgeon for the illustrative surgical images in Section 7.4. The opinions can be employed to give a guidance for developing an automatic algorithm that produces illustrative surgical images.

## 8. Conclusions and Future Work

In this paper, we produced illustrated surgical images to prove their ability to reduce the negative emotional responses engendered by photographic surgical images. We executed emotion recognition processes on 40 participants to compare their emotional responses to photographic and illustrated surgical images. The emotional responses were estimated in a bi-modal approach: a deep learning-based emotion recognition model from EEG biosignals was combined with a 9-point metric user annotation. From the results, we conclude that illustrated surgical images indeed capable of reducing the negative emotions of participants.

In our future research, we will study relevant methods to create appropriate illustrated images. We will consult with experts including surgeons and pathologists to enrich the illustration schemes on surgical images. We will also examine the illustrated surgical images from experts to improve the quality of the illustrations. These approaches will help developing an automatic algorithm for generating illustrated surgical images that satisfy both ordinary people and experts.

## Figures and Tables

**Table 1:** Gender and age distribution of the participants.

	Total	Gender		Age		
		Female	Male	20 s	30 s	>40
group1	20	10	10	13	6	1
group2	20	10	10	12	8	0

**Table 2:** Results of our experiment valence and arousal values in EEG-estimated matrix are rearranged to (−1∼1) scale.

EEG-Estimated						User-Annotated					
Photographic Surgical			Illustrated Surgical			Photographic Surgical			Illustrated Surgical		
Part. No.	Val.	Arou.	Part. No.	Val.	Arou.	Part. No.	Val.	Arou.	Part. No.	Val.	Arou.
01	−0.86	0.34	21	−0.68	0.21	01	2	6	21	2	3
02	−0.91	0.64	22	−0.39	0.24	02	1	9	22	4	5
03	−0.76	0.47	23	−0.54	0.09	03	2	7	23	1	4
04	−0.85	0.55	24	−0.48	0.19	04	3	7	24	4	5
05	−0.73	0.49	25	−0.45	0.17	05	3	7	25	4	4
06	−0.93	0.51	26	−0.53	0.18	06	1	7	26	3	5
07	−0.81	0.58	27	−0.59	0.13	07	2	7	27	2	5
08	−0.84	0.63	28	−0.52	0.25	08	1	8	28	3	6
09	−0.85	0.65	29	−0.63	0.21	09	1	8	29	2	5
10	−0.86	0.61	30	−0.61	0.311	10	1	9	30	3	6
11	−0.84	0.59	31	−0.43	0.26	11	3	8	31	3	6
12	−0.88	0.52	32	−0.47	0.24	12	1	7	32	5	6
13	−0.92	0.51	33	−0.51	0.23	13	1	7	33	3	5
14	−0.78	0.48	34	−0.56	0.28	14	2	5	34	3	5
15	−0.83	0.53	35	−0.64	0.27	15	1	7	35	3	6
16	−0.89	0.55	36	−0.65	0.26	16	1	7	36	1	7
17	−0.79	0.59	37	−0.59	0.23	17	1	7	37	3	5
18	−0.80	0.53	38	−0.58	0.26	18	2	7	38	3	6
19	−0.76	0.51	39	−0.52	0.31	19	4	6	39	2	6
20	−0.74	0.55	40	−0.61	0.33	20	2	7	40	3	6

**Table 3:** *p* values for *t*-test between photographic and illustrated surgical images.

	EEG-Estimated		User-Annotated	
	Photographic Surgical	Illustrated Surgical	Photographic Surgical	Illustrated Surgical
	(group1)	(group2)	(group1)	(group2)
valence	$2.27\times 10^{-15}$		$7.6\times 10^{-4}$	
arousal	$2.07\times 10^{-17}$		$2.2\times 10^{-7}$	

**Table 4:** *p* values for *t*-test between EEG-estimated and user-annotated valence and arousal.

	Photographic Surgical (group1)		Illustrated Surgical (group2)	
	EEG-Estimated	User-Annotated	EEG-Estimated	User-Annotated
valence	0.72134		0.844513	
arousal	0.942176		0.007345	

**Table 5:** Cohen’s *d* values to measure the difference in emotional reaction between photographic and illustrated surgical images.

	EEG-Estimated		User-Annotated	
	Photographic Surgical	Illustrated Surgical	Photographic Surgical	Illustrated Surgical
	(group1)	(group2)	(group1)	(group2)
valence	(i) 1.78		(ii) 1.01	
arousal	(iii) 1.82		(iv) 1.37	

**Table 6:** Cohen’s *d* values to measure the difference of the emotional reaction estimation methods: EEG-biosignal and user-annotation.

	Photographic Surgical (group1)		Illustrated Surgical (group2)	
	EEG-Estimated	User-Annotated	EEG-Estimated	User-Annotated
valence	(i) 0.12		(ii) 0.06	
arousal	(iii) 0.23		(iv) 0.64	

**Table 7:** Comparison to existing models that recognizes valence and arousal using DEAP dataset.

Existing Models	Classifier	Accuracy (%)	
		Valence	Arousal
Khosrowabadi et al. 2014 [14]	RNN	71.43	70.83
Alhagry et al. 2017 [16]	LSTM RNN	85.00	85.00
Li et al. 2017 [17]	CRNN	72.06	74.12
Salama et al. 2018 [8]	3D CNN	87.44	88.49
Xing et al. 2019 [18]	LSTM	81.10	74.38
Ours	multi-column	90.01	90.65
